# Gut microbiota contributes to the methionine metabolism in host

**DOI:** 10.3389/fmicb.2022.1065668

**Published:** 2022-12-22

**Authors:** Xiaoyan Wu, Ziyi Han, Bingnan Liu, Dongming Yu, Jing Sun, Liangpeng Ge, Wenjie Tang, Shaojuan Liu

**Affiliations:** ^1^State Key Laboratory for Conservation and Utilization of Subtropical Agro-Bioresources, Guangdong Laboratory of Lingnan Modern Agriculture, National Engineering Research Center for Breeding Swine Industry, Guangdong Provincial Key Laboratory of Animal Nutrition Control, College of Animal Science, South China Agricultural University, Guangzhou, China; ^2^Chongqing Academy of Animal Sciences, Chongqing, China; ^3^Animal Breeding and Genetics Key Laboratory of Sichuan Province, Sichuan Animal Science Academy, Chengdu, China; ^4^Livestock and Poultry Biological Products Key Laboratory of Sichuan Province, Sichuan Animtech Feed Co., Ltd., Chengdu, China

**Keywords:** gut microbiota, methionine, SAM, hCY, germ-free pigs

## Abstract

Methionine (Met) metabolism provides methyl groups for many important physiological processes and is implicated in multiple inflammatory diseases associated with the disrupted intestinal microbiota; nevertheless, whether intestinal microbiota determines Met metabolism in the host remains largely unknown. Here, we found that gut microbiota is responsible for host Met metabolism by using various animal models, including germ-free (GF) pigs and mice. Specifically, the Met levels are elevated in both GF pigs and GF mice that mainly metabolized to S-adenosine methionine (SAM) in the liver. Furthermore, antibiotic clearance experiments demonstrate that the loss of certain ampicillin- or neomycin-sensitive gut microbiota causes decreased Met in murine colon. Overall, our study suggests that gut microbiota mediates Met metabolism in the host and is a prospective target for the treatment of Met metabolism-related diseases.

## Introduction

Methionine (Met) is one of the essential amino acids that could participate in the biosynthesis of various nitrogen-containing substances (Liu et al., 2021). Indeed, Met metabolism in the host mainly consists of four metabolic pathways surrounding the Met-homocysteine (hCy) cycle (Yin et al., 2018; Xu et al., 2020). The host Met-hCy cycle (hereinafter referred to as the Met cycle) is associated with transmethylation and the metabolism of amino acids such as serine (Ser), glycine (Gly), glutathione (GSH), and taurine (Tau; Xu et al., 2020). Specifically, Met reacts with ATP under the catalysis of methionine adenosyltransferase (MAT) to generate S-adenosine methionine (SAM), which then provides a methyl group to generate S-adenosine homocysteine (SAH; Clare et al., 2019). Homocysteine (hCy) generated by S-adenosylhomocysteine hydrolase (SAHH) is then remethylated by N5-methyltetrahydrofolate (CH_3_-THF) to generate Met, and vitamin B_12_ serves as a key coenzyme in this process (Lam et al., 2021). Of note, the Met cycle ensures Met level and the supply of methyl-donor SAM in liver; therefore, modulating Met cycle has therapeutic implications for many diseases, for example, alcoholic liver disease (ALD; Kharbanda, 2013). Moreover, the Met cycle in tumors has also received attention. For example, tumor initiation of lung cancer stem cells consumes large amounts of Met for histone methylation, resulting in Met depletion and exogenous Met dependence (Wang et al., 2019). Met cycle is also disrupted in liver tumors and is often associated with poor prognosis (Xu et al., 2020). Therefore, it is meaningful to fine-tune the Met metabolism in host. However, the factors affecting the Met metabolism are still unclear.

The gut has a complex microorganism community including bacteria, fungi, and viruses, which the composition is determined by natural colonization and subsequent environmental factors (Cani, 2018). Gut microbes play particularly important roles in regulating homeostasis by absorbing nutrients, producing metabolites, and cooperating or competing with other microorganisms in the intestinal tract (Osbelt et al., 2021). It is worth noting that the impact of the gut microbiota on host is not limited in gut, as supported by the findings of the gut-brain axis (Cryan et al., 2019), gut-lung axis (Dang and Marsland, 2019), and gut-liver axis (Tripathi et al., 2018). A study revealed that patients with quiescent inflammatory bowel disease (IBD) fatigue had reduced serum metabolites like Met, Ser and sarcosine (Sar), and decreased short-chain fatty acids (SCFAs)-producing gut microbiota such as *Faecalibacterium prausnitzii* (Borren et al., 2021). In a controlled study of Parkinson’s disease (PD), significant differences in the trajectories of Met and homoserine were found (Hertel et al., 2019). And the PD-associated gut microbiome analysis revealed increased abundance of *Akkermansia muciniphila* (*A. muciniphila*) and *Bilophila wadsworthia* involved in sulfur metabolism (Hertel et al., 2019). Additionally, *A. muciniphila* contributed more than 70% of Met secretion potential (Hertel et al., 2019), indicating the interaction between the gut microbiota and host Met cycle. Nevertheless, whether Met metabolism in host is related to the gut microbiota remains enigmatic.

Given the importance of Met metabolism in inflammation, cancer, and neurological diseases and the impact of microbial-host interactions on host physiology, biochemistry, and immune status, an in-depth analysis of gut microbial and host Met metabolism has profound implications. Microbiota-targeted interventions mainly include antibiotic treatment, probiotic addition, and fecal microbiota transplantation (Sun and Shen, 2018). Among them, antibiotic-induced intestinal dysbiosis is a common method that can lead to drastic changes in the intestinal microbiota (Andremont et al., 2021). Notably, germ-free (GF) animal models are currently important models for studying microbiome-host interactions. Unlike pharmacological interventions such as antibiotics, GF animals achieve true “sterility” and have distinct characteristics from specific pathogen-free (SPF) animals in gut development and/or immune system (Uzbay, 2019). Therefore, GF animals are valuable experimental models in cancer and metabolism-related diseases. Moreover, studies of Met metabolism have typically used *in vitro* cell or tissue cultures and validated in rodents (Wang et al., 2019; Xu et al., 2020). Genetically inbred mice in rodents are common experimental models that help scientists accomplish many studies, but they are not ideal models of the human immune system because of the differences in immune systems of humans and mice caused by evolutionary branches (Pulendran and Davis, 2020). Therefore, the GF pig model adopted in this paper may help to provide a more valuable reference.

As experimental subjects, pigs are ideal large animal models for studying nutrition or disease in pigs and humans due to their high similarity to humans in terms of anatomy, physiology, and immune system (Miller and Ullrey, 1987; Itoh et al., 2019). The GF pig model is therefore ideal for studying gut microbe-host Met metabolism. Importantly, dietary Met plays an important role in animal nutrition and is the second/third limiting amino acid in pig and piglet diets (Yang et al., 2021). Since Met is beneficial for the growth and development of pigs, most relevant studies have focused on the effects of dietary Met or Met-restricted diets on swine meat quality, growth performance, and intestinal function (Zeitz et al., 2019; Gondret et al., 2021; Yang et al., 2021). However, there are few studies concerning Met metabolism in pigs. Currently, only limited studies on plasma Met metabolism profiles and mammalian lifespan have been retrieved focusing on the relationship between porcine plasma Met metabolism and lifespan (Mota-Martorell et al., 2021).

Therefore, we used GF pig as an experimental model to explore the impact of gut microbiota on host Met metabolism that contributes to map the Met metabolism profile of the pig gut microbiome. We then explored the link between gut microbiota and host Met metabolism in GF mice and mice treated with different antibiotics, further expanding the generalizability of the experimental results and potentially providing new insights into Met metabolism-related diseases.

## Materials and methods

### Animal acquisition and breeding

#### GF and SPF pigs

GF pigs and SPF pigs were harvested by uterine dissection and then transferred to sterile feeding isolators (DOSSY Experimental Animals Co., Ltd., Chengdu, China) for decontamination. Next, the piglets were taken to rearing isolators (Class Biologically Clean Ltd., Madison, Wisconsin, United States; Qi et al., 2021; Zhou et al., 2021). The isolator operates in strict accordance with operating procedures within the barrier facilities.

The feeding conditions of the two groups of experimental animals are the same, and the specific steps refer to our previous experiments (Liu et al., 2022). For the first 21 days, they were fed Co60 γ-irradiated 4.8%-fat sterile milk powder (Jiangsu Anyou Group China) diluted with sterile water (Sun et al., 2018; Liu et al., 2022). The basal diet for pigs from day 22 was provided by the Institute of Animal Nutrition, Sichuan Agricultural University (Chengdu, China). In this study, pigs were obtained from the Technical Engineering Center for the Development and Utilization of Medical Animal Resources (Chongqing, China). All experimental protocols were approved and carried out under the guidance of the Laboratory Animal Ethics Committee of South China Agricultural University [Guangzhou, China; permit number SYXK (Guangdong) 2019–0136].

Eleven GF pigs were randomly selected, five of which were kept under GF conditions (GF pigs) and their anal swabs were collected weekly for microbial testing. Another six pigs served as controls (SPF pigs) by transplanting maternal feces. Control pigs were continuously orally administered fecal bacteria suspension (1 ml/d) for 3 days at 7 days of age and kept in sterile isolators (Liu et al., 2022).

**Figure 1:**
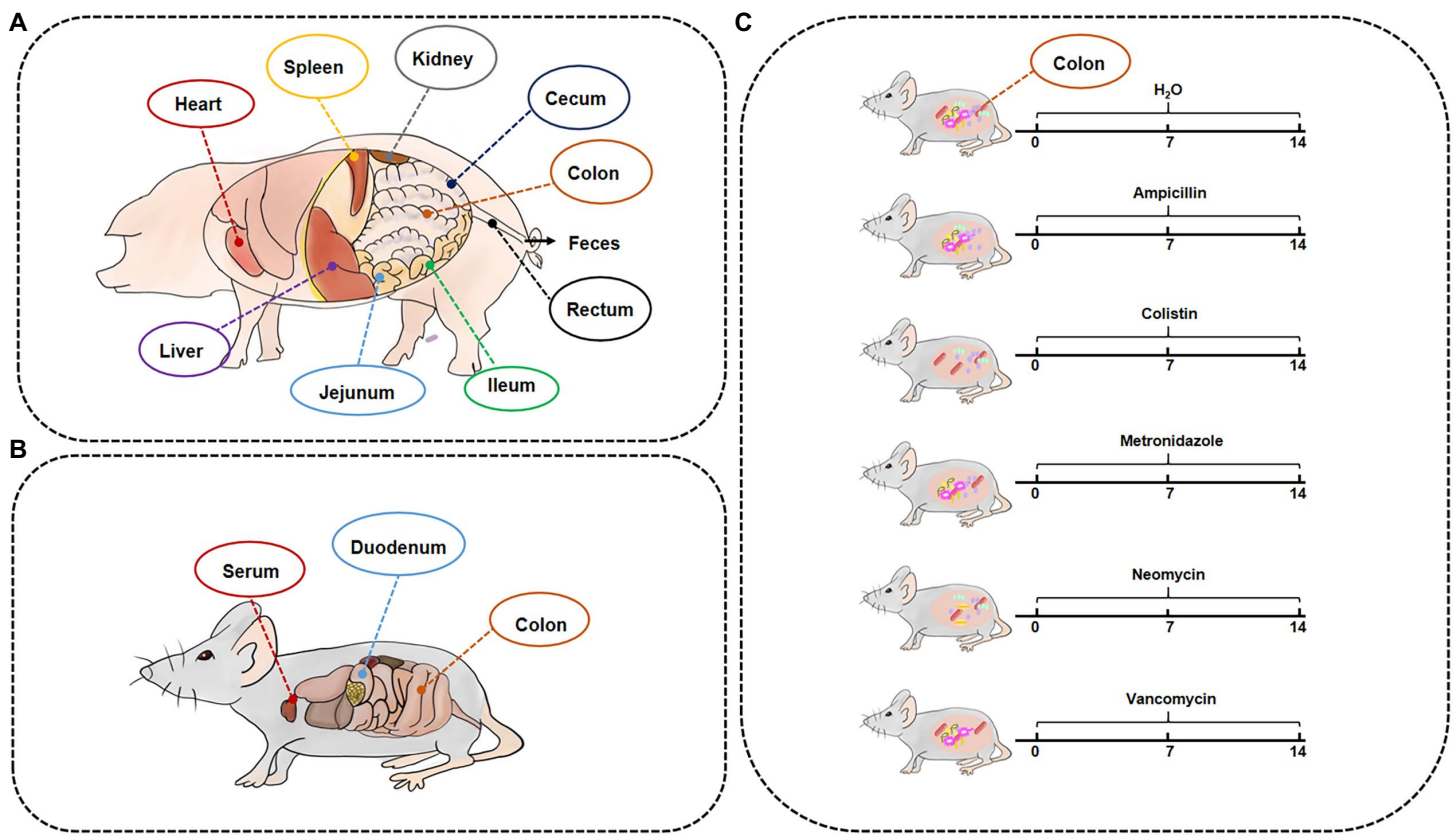
Timeline of experimental processing and sample collection information. The samples of GF/SPF pigs **(A)** and mice **(B)**. **(C)** SPF mice were treated by different antibiotics for 2 weeks.

For sample collection, on the last day of the experimental period, two groups of pigs were subjected to respiratory anesthesia with isoflurane after urine collection, and other samples were collected on a clean bench after blood collection to ensure that there was no bacterial contamination throughout the entire sampling process. Liver, bile, spleen, kidney, heart, and different intestinal segments and their contents were collected, including jejunum, ileum, cecum, colon, jejunum contents, cecal contents, colonic contents, rectal contents, and feces (Figure 1A). Samples were quickly frozen in liquid nitrogen and stored in an ultra-low temperature freezer at −80°C.

#### GF/SPF and antibiotic-treated mice

GF and SPF Kunming mice were bred at the Third Military Medical University (Chongqing, China). ICR mice were purchased from Liaoning Changsheng Biotechnology Co., Ltd. (Liaoning, China). GF mice were housed in sterile isolators and SPF Kunming and ICR mice were housed in sterile individual animal colonies (temperature, 25 ± 5°C; relative humidity, 55 ± 5%; dark light alternated every 12 h) with free access to drinking water and standard rodent chow.

For the Met cycle metabolite analysis experiments, GF (GF group, 4 weeks old, *n* = 10 males and 10 females) and SPF (SPF group, 4 weeks old, *n* = 10 males and 10 females) Kunming mice were treated with the same diet for 4 weeks, then were euthanized by CO_2_ asphyxiation, and blood, duodenum, and colon were collected (Figure 1B).

To establish an antibiotic-treated mouse model, 6-week-old female ICR mice (*n* = 12) were supplemented with ampicillin (MB1407, 1 mg/ml, meilunbio), colistin (MB1064, 1 mg/ml, meilunbio), metronidazole (B300250, 1 mg/ml, Aladdin), neomycin (MB1716, 1 mg/ml, meilunbio), and vancomycin (V301569, 0.5 mg/ml, Aladdin) in drinking water for 2 weeks. Mice were then euthanized by CO_2_ asphyxiation and colon samples were collected (Figure 1C).

### Standard and sample preparation

The key metabolites in the Met cycle involved in this study are Met, SAM, SAH, and hCY. Accurately weighed 1.0 mg of these metabolites and dissolved each standard in 10 ml methanol–water (50:50, V/V) to obtain a 100 μg/ml stock solution. The standard solution was then serially diluted to 1,000, 500, 100, 10, 1, 0.5, 0.1, 0.01, 0.001 ng/ml. All metabolites were quantified by external standard method, and all standard solutions were stored at −20°C. The processing of tissue samples and fluid samples such as serum, urine, and bile was carried out according to our previous experiments (Liu et al., 2022), and stored at −20°C prior to UPLC-Orbitrap-MS/MS analysis.

### UPLC-Orbitrap-MS/MS conditions

Met cycle-related metabolites were separated by Thermo Fisher Scientific UPLC system (Dionex UltiMate 3,000) with a C1s Hypersil Gold column (1.9 μm, 100 mm × 2.1 mm; Thermo Scientific). The main parameters and gradient elution procedure were based on our previous experiments (Liu et al., 2022).

### Identification of metabolites

Metabolite identification refers to our previous experiments (Liu et al., 2022). Briefly, information such as retention time, mass (m/z), peak and fragment ion MS or MS/MS intensity could be obtained when the RAW file is opened with Xcalibur. The mass list of each MS scan was generated by the peak detection and chromatographic builder in the original data file, and then the chromatogram was built.

### Data analysis and statistics

Xcalibur converted data in an instrument-specific format (*.raw) to a common data format (.XLS) that displays information on metabolites, including calculated amounts, retention times, and peak areas. The raw data can be accessed by the Metabolights repository,^1^ with the identifier MTBLS6154 (Haug et al., 2020).

All data are shown as mean ± SEM. Data between two groups were analyzed by (unpaired *t*-test, Prism 6.0) if the data exhibited Gaussian distribution and equal variance, and by (Welch-corrected unpaired *t*-test, Prism 6.0) if the data exhibited (Gaussian distribution but unequal variances). If the data were not normally distributed, a nonparametric test (Mann–Whitney U test, Prism 6.0) was performed. The Gaussian distribution of the data was analyzed by (D’Agostino-Pearson comprehensive normality test, Prism 6.0) and/or (Kolmogorov–Smirnov test, Prism 6.0). Data were analyzed for variance by (Brown-Forsythe test, Prism 6.0). Significant differences among 3 or more groups were passed (Bonferroni’s multiple comparison test, one-way ANOVA assessment, and multiple *t*-test, Prism 6.0). *p* < 0.05 (*), *p* < 0.01 (**), *p* < 0.001 (***), and *p* < 0.0001 (****) were considered statistically.

## Results

### Gut microbiota alters the met cycle of pig gut

**Figure 2:**
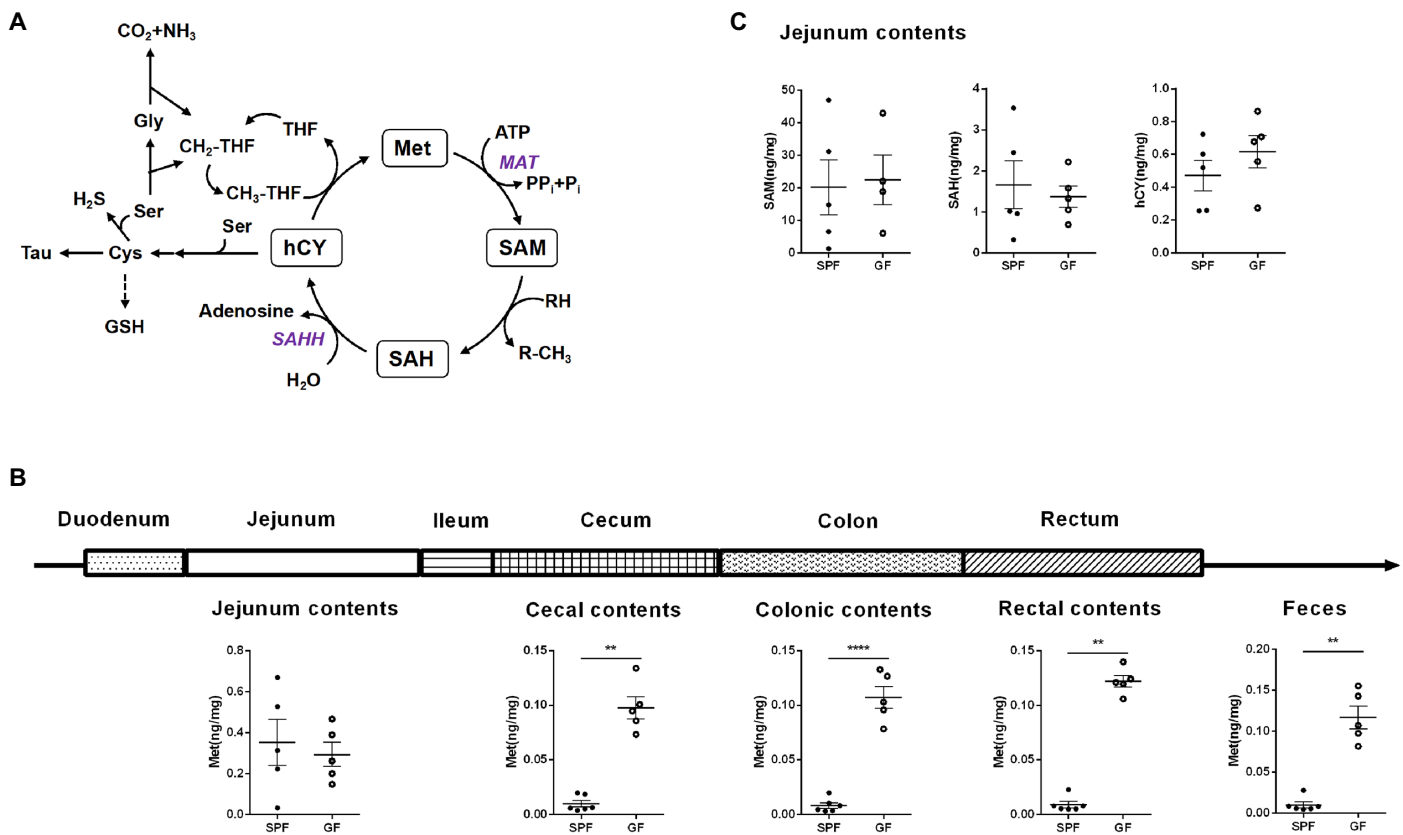
Gut microbiota alters the Met metabolism of pig gut. **(A)** Met cycle. Met consumes ATP under the catalysis of MAT to generate SAM, SAM provides methyl group to generate SAH, SAH generates hCY under the action of SAHH, and hCY obtains methyl group through CH_3_-THF in the folic acid cycle to generate Met. hCY participates in the generation of Cys, which, in turn, generates GSH or Tau. **(B)** Met content in different intestinal contents of GF pigs (*n* = 5) and SPF pigs (*n* = 6). **(C)** Contents of SAM, SAH, and hCY in the jejunum contents of GF pigs (*n* = 5) and SPF pigs (*n* = 6). Data were analyzed by unpaired *t*-test and represented as means ± SEM. *p* < 0.05 (*), *p* < 0.01 (**), *p* < 0.001 (***), and *p* < 0.0001 (****). Met: Methionine; SAH: S-adenosine homocysteine; SAM: S-adenosine methionine; hCY: homocysteine; Ser: Serine; Cys: Cysteine; Tau: Taurine; GSH: Glutathione; Tau: Taurine; Gly: Glycine; THF: Tetrahydrofolate; MAT: Methionine adenosyltransferase; SAHH: S-adenosylhomocysteine hydrolase.

In this study, we collected different intestinal contents of GF pigs and analyzed by UPLC (Figure 1A). Four key metabolites in the Met cycle were detected, including Met, SAM, SAH, and hCY (Figure 2A). Of note, SAM, generated by Met metabolism, is an important methyl donor that cooperates with the methylation *in vivo*, such as histone methylation (Yu et al., 2019). HCY from SAH deadenosine may regulate the one-carbon metabolism of Ser and Gly, the folate cycle of THF, or the generation of cysteine (Cys) to generate GSH or Tau (Selhub, 1999; Mentch et al., 2015; Jakubowski, 2019). As a result, Met level was significantly increased by almost 10-fold in the gut contents (including cecum, colon, rectal contents, and feces) of GF pigs compared to the control group (SPF), with the exception of the jejunum contents (Figure 2B). SAM, SAH, and hCY were not significantly altered in the jejunum contents of GF pigs (Figure 2C) that may be present at very low levels even below the detection limit in other intestinal contents. The experimental results showed that the absence of the gut microbiota led to a significant increase of Met levels in intestinal contents, suggesting that gut microbiota was involved in the regulation of the Met cycle in pig intestines.

### Gut microbiota regulates the met cycle in different intestinal segments of pigs

**Figure 3:**
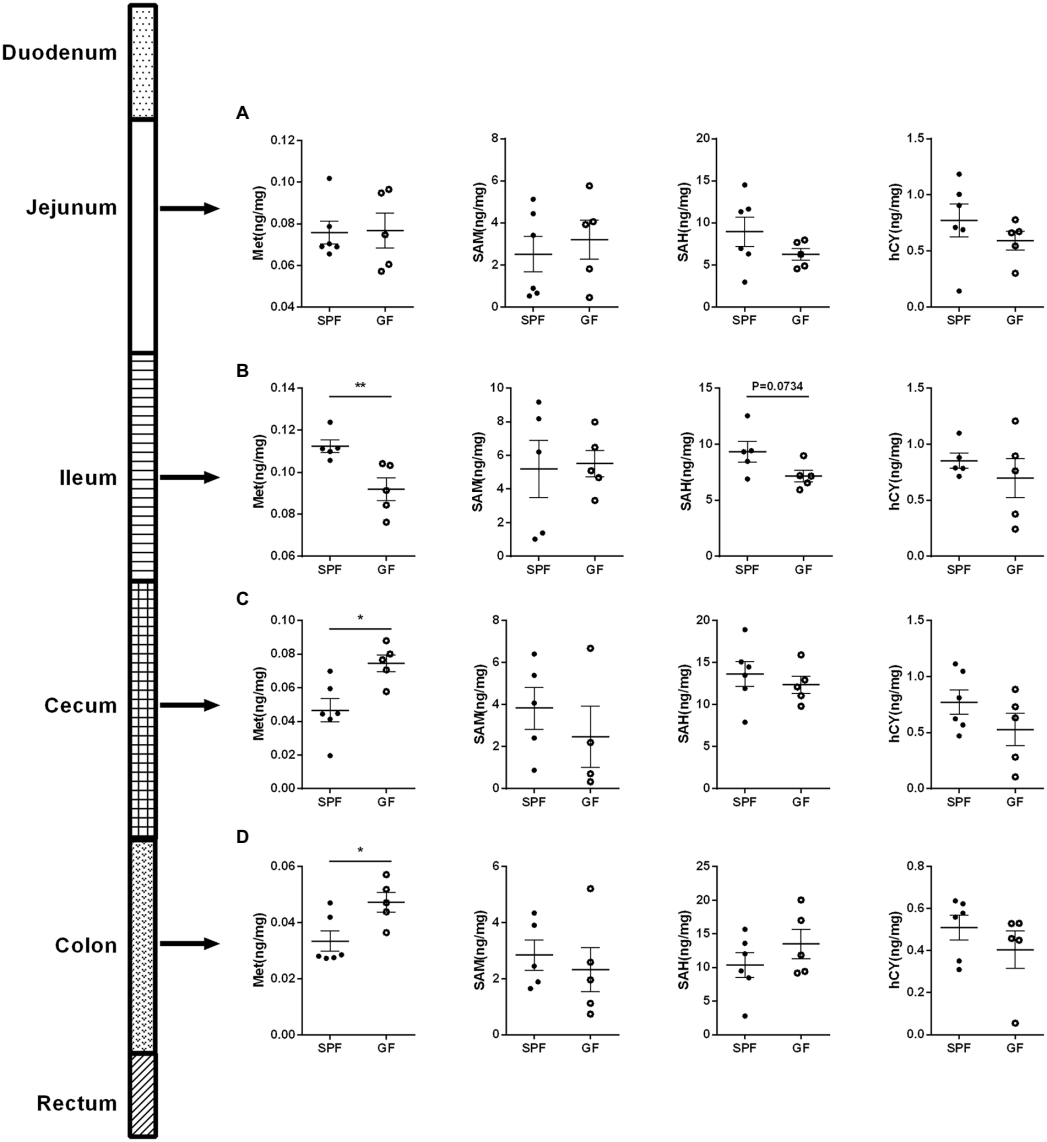
Gut microbiota regulates the Met metabolism in different intestinal segments of pigs. Contents of Met, SAM, SAH, hCY in **(A)** jejunum, **(B)** ileum, **(C)** cecum, and **(D)** colon of GF pigs (*n* = 5) and SPF pigs (*n* = 6). Data were analyzed by unpaired *t*-test and represented as means ± SEM. *p* < 0.05 (*), *p* < 0.01 (**), *p* < 0.001 (***), and *p* < 0.0001 (****). Met: Methionine; SAH: S-adenosine homocysteine; SAM: S-adenosine methionine; hCY: homocysteine.

About 25% of the body’s trans-methylation and trans-sulfurization reactions occurred in the gastrointestinal tract, and 65% of the visceral first-pass effect of Cys occurred in the intestine (Bauchart-Thevret et al., 2009), indicating that intestinal absorption was crucial to Met cycle. Therefore, we examined Met cycle-associated metabolites in different intestinal segments. There was no significance in the four key metabolites in the jejunum (Figure 3A). Compared with SPF group, Met in the ileum of GF group was significantly decreased, and SAH showed a downward trend (Figure 3B). In the cecum and colon, Met was increased in GF group compared to SPF group, but SAM, SAH, and hCY were not significantly different (Figures 3C,D). Together, the increase of Met in GF pigs was mainly occurred in the large intestinal segment.

### Gut microbiota remodels the met cycle in extraintestinal tissue and circulation system of pigs

The Met metabolites are absorbed by the intestinal epithelium and penetrate the basement membrane and then conducted on the extraintestinal tissues to further exert their effects through blood circulation and other means (Kharbanda, 2013; Xu et al., 2020). In addition, the liver is an important organ for Met metabolism that converts Met to SAM by MAT1A (Methionine adenosyltransferase 1A; Lu and Mato, 2012). Met-related metabolites are perturbed in both cardiovascular disease (CVD) and chronic kidney disease (CKD; Xiao et al., 2015; Yang et al., 2016), and the kidneys are involved in SAH metabolism (Garibotto et al., 2009).

**Figure 4:**
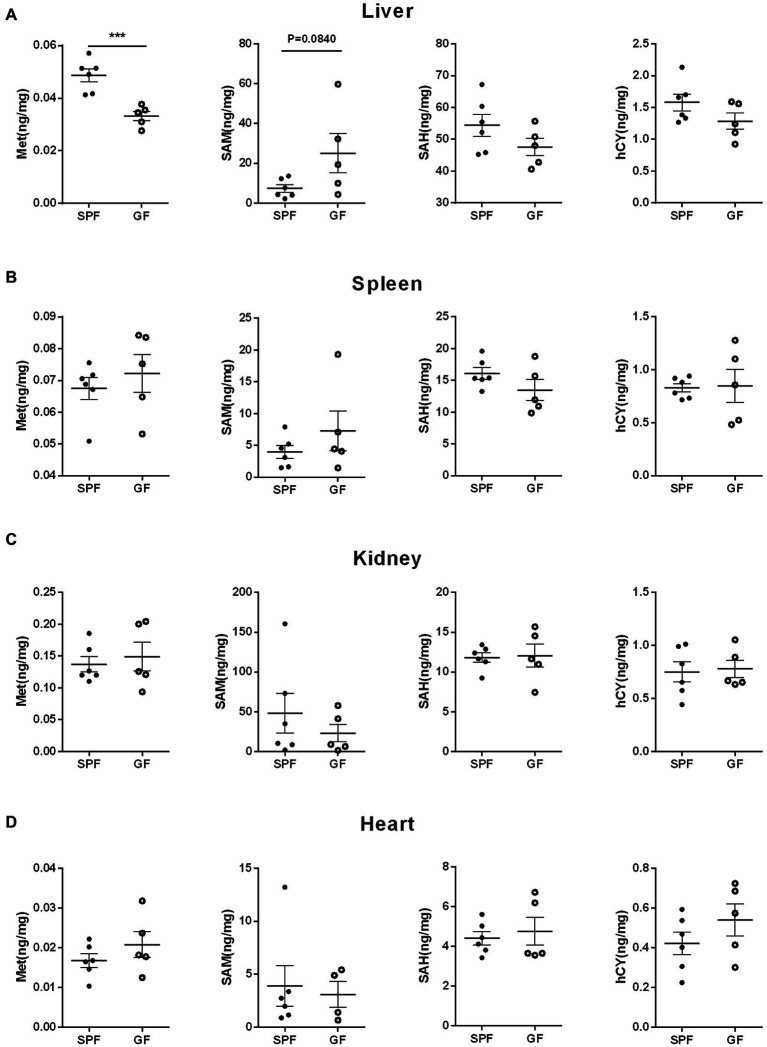
Gut microbiota remodels the Met metabolism in porcine extraintestinal tissue by gut microbiota. Contents of Met, SAM, SAH, hCY in **(A)** liver, **(B)** spleen, **(C)** kidney, **(D)** heart of GF pigs (*n* = 5) and SPF pigs (*n* = 6). Data were analyzed by unpaired *t*-test and represented as means ± SEM. *p* < 0.05 (*), *p* < 0.01 (**), *p* < 0.001 (***), and *p* < 0.0001 (****). Met: Methionine; SAH: S-adenosine homocysteine; SAM: S-adenosine methionine; hCY: homocysteine.

**Figure 5:**
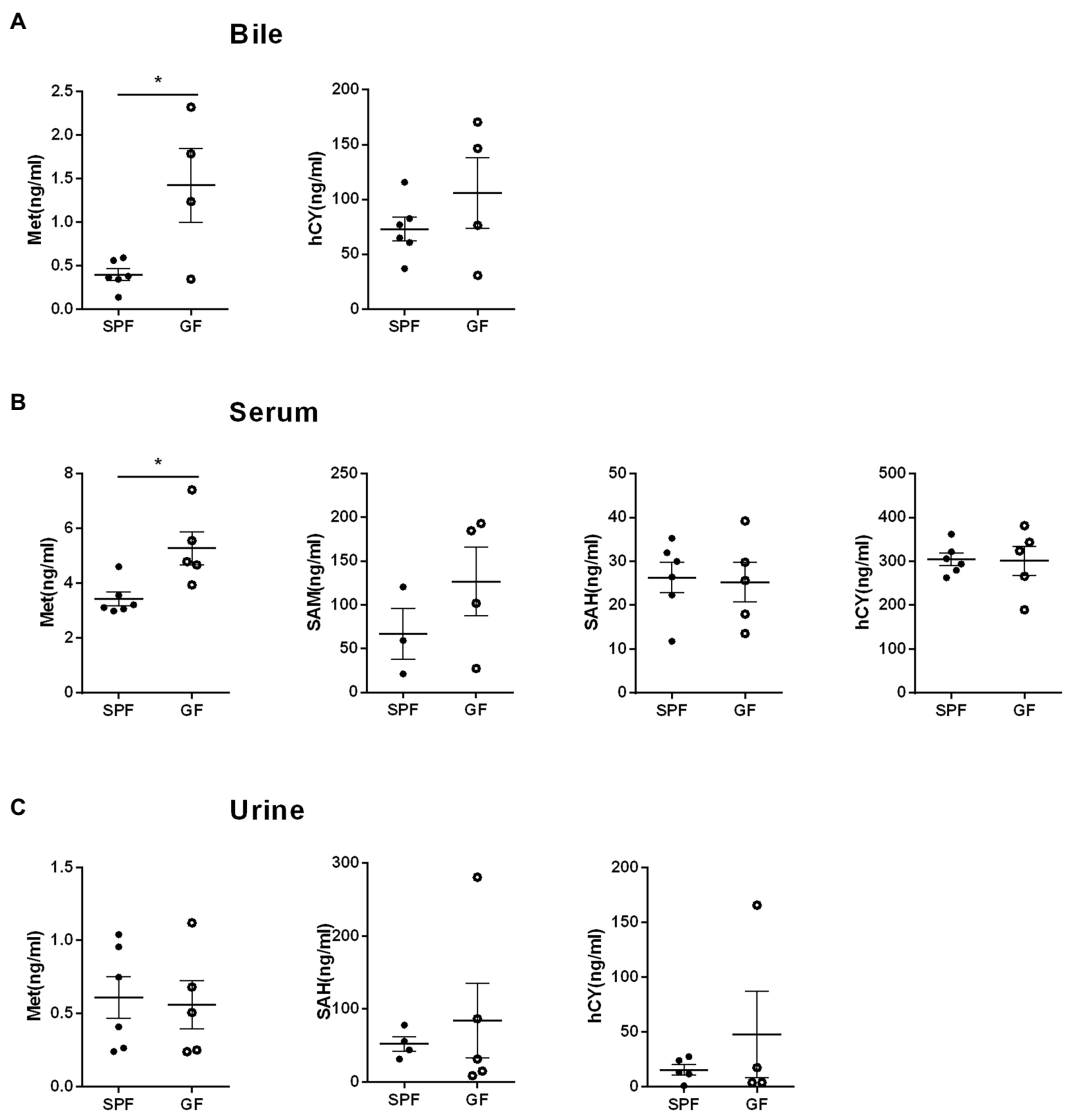
Gut microbiota modulates Met metabolism in porcine circulation system. **(A)** Met and hCY levels in bile. **(B)** Met, SAM, SAH, hCY levels in serum. **(C)** Met, SAH, hCY levels in urine of GF pigs (*n* = 5) and SPF pigs (*n* = 6). Data were analyzed by unpaired *t*-test and represented as means ± SEM. *p* < 0.05 (*), *p* < 0.01 (**), *p* < 0.001 (***), and *p* < 0.0001 (****). Met: Methionine; SAH: S-adenosine homocysteine; SAM: S-adenosine methionine; hCY: homocysteine.

**Figure 6:**
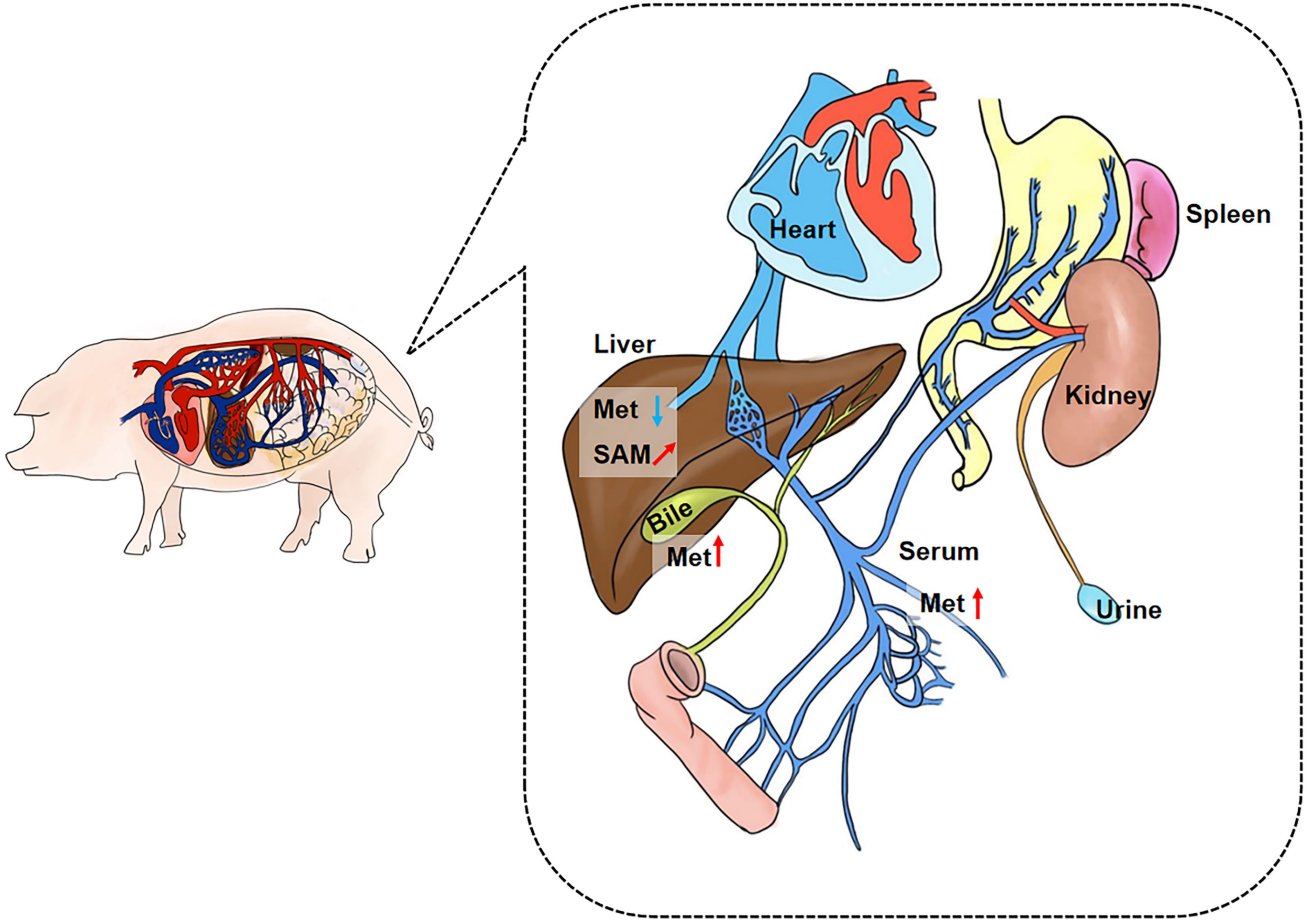
Summary plot of Met metabolic changes in GF porcine parenteral tissue and parenteral circulation. Met was increased in the overall circulation in GF pigs, and the elevated Met was mainly utilized in liver. Met: Methionine; SAH: S-adenosine homocysteine; SAM: S-adenosine methionine. *(“↗”arrows represent increasing trend, “⬆” arrows represent increases, and “⬇” arrows represent decreases.)

We collected extraintestinal tissue samples from GF pigs and SPF pigs, including liver, spleen, kidney, and heart, and detected and analyzed the key metabolites of the Met cycle, respectively, by UPLC-Orbitrap-MS/MS. The results showed that Met in the liver of GF pigs decreased and SAM tended to increase (Figure 4A). There were no significant differences in Met, SAM, SAH, and hCY in spleen (Figure 4B), kidney (Figure 4C), and heart (Figure 4D) between GF and SPF groups. Analogously, blood, bile, and urine in the circulation system of both groups (GF and SPF) were detected by UPLC-Orbitrap-MS/MS. The results illustrated that Met was elevated in bile (Figure 5A) and serum (Figure 5B) but was no significant difference in urine (Figure 5C) in GF group. This result suggested that the lack of gut microbiota led to the increase of Met in the circulatory system, which was converted to SAM in the liver (Figure 6).

### Gut microbiota modulates the met cycle of mice

**Figure 7:**
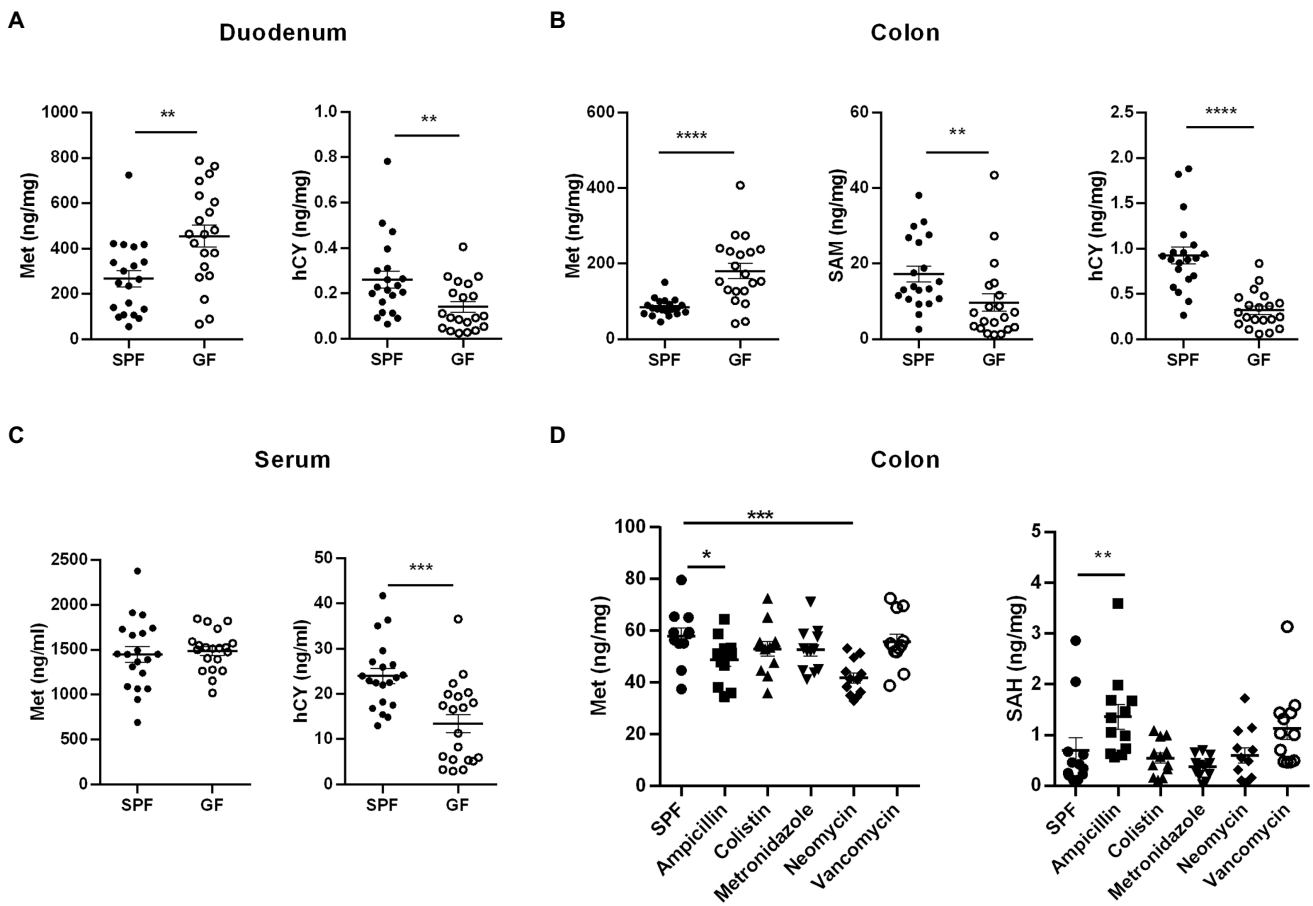
Influence of gut microbiota on Met metabolism in mice. **(A)** Met and hCY contents in duodenum of GF mice and SPF mice (*n* = 20). **(B)** Met, SAM, and hCY contents in colons of GF mice and SPF mice (*n* = 20). **(C)** Serum Met and hCY levels in GF mice and SPF mice (*n* = 20). **(D)** Met and SAH contents in colon of SPF mice (*n* = 12) after different antibiotic treatments. Data were analyzed by unpaired *t*-test (two groups) and one-way ANOVA assessment (>two groups), and represented as means ± SEM. *p* < 0.05 (*), *p* < 0.01 (**), *p* < 0.001 (***), and *p* < 0.0001 (****). Met: Methionine; SAH: S-adenosine homocysteine; SAM: S-adenosine methionine; hCY: homocysteine.

By collecting samples from GF pigs and SPF pigs, we detected Met cycle-metabolites in the intestinal contents, different intestinal segments, extraintestinal tissues, and the circulation system. It was found that the absence of gut microbiota resulted in an overall increase in circulating Met and the elevated Met might be primarily utilized in liver. Next, we sought to translate the involvement of gut microbiota on Met metabolism to rodents. We collected duodenum, colon, and serum samples from GF and SPF mice and examined key Met cycle metabolites. Met was significantly increased and hCY was significantly decreased in the duodenum and colon of GF mice (Figures 7A,B). SAM in the colon of GF mice was significantly decreased (Figure 7B), which was consistent with the trend in pigs. There was no significant difference on Met in the serum of GF mice, but hCY was significantly reduced (Figure 7C). Thus, the findings further confirmed that the gut microbiota can modulate the Met metabolism and the absence of gut microbiota resulted in elevated Met and lower downstream SAM and/or hCY.

In addition to host metabolism of Met, it has been demonstrated the existence of microbial metabolism of Met and Cys (Das et al., 2021). For example, gut microbes use cysteine desulfurase (CDS) to catalyze the synthesis of H_2_S from Cys (Wang et al., 2019). In the intestinal flora, Gram-positive *Streptococcus*, *Clostridium*, *Staphylococcus aureus*, *Mycobacterium tuberculosis*, Gram-negative *Klebsiella*, *Enterobacter*, *Salmonella, Akkermansia* spp., and *Desulfovibrio* are involved in the degradation of Cys (Carbonero et al., 2012). In the lower gastrointestinal tract, taurine, and isethionate are substrates for microbial metabolism, and microorganisms metabolize these organic sulfonates with the assistance of electron carriers to produce sulfide, ammonia, and other products (Laue et al., 1997). Therefore, to determine the types of microbes played pivotal roles in the regulation of host’s Met metabolism, we constructed a model of microbe deletion by treating mice with different antibiotics in drinking water for 2 weeks. The results showed decreased Met and increased SAH in colon of ampicillin-treated mice (Figure 7D). Neomycin treatment caused decreased colonic Met levels (Figure 7D). However, colonic Met and SAH were not significantly different between colistin, metronidazole, and vancomycin-treated mice (Figure 7D). Therefore, we speculated that part of the ampicillin- and/or neomycin-sensitive gut microbiota has taken a major role in regulating the Met cycle in host.

## Discussion

**Figure 8:**
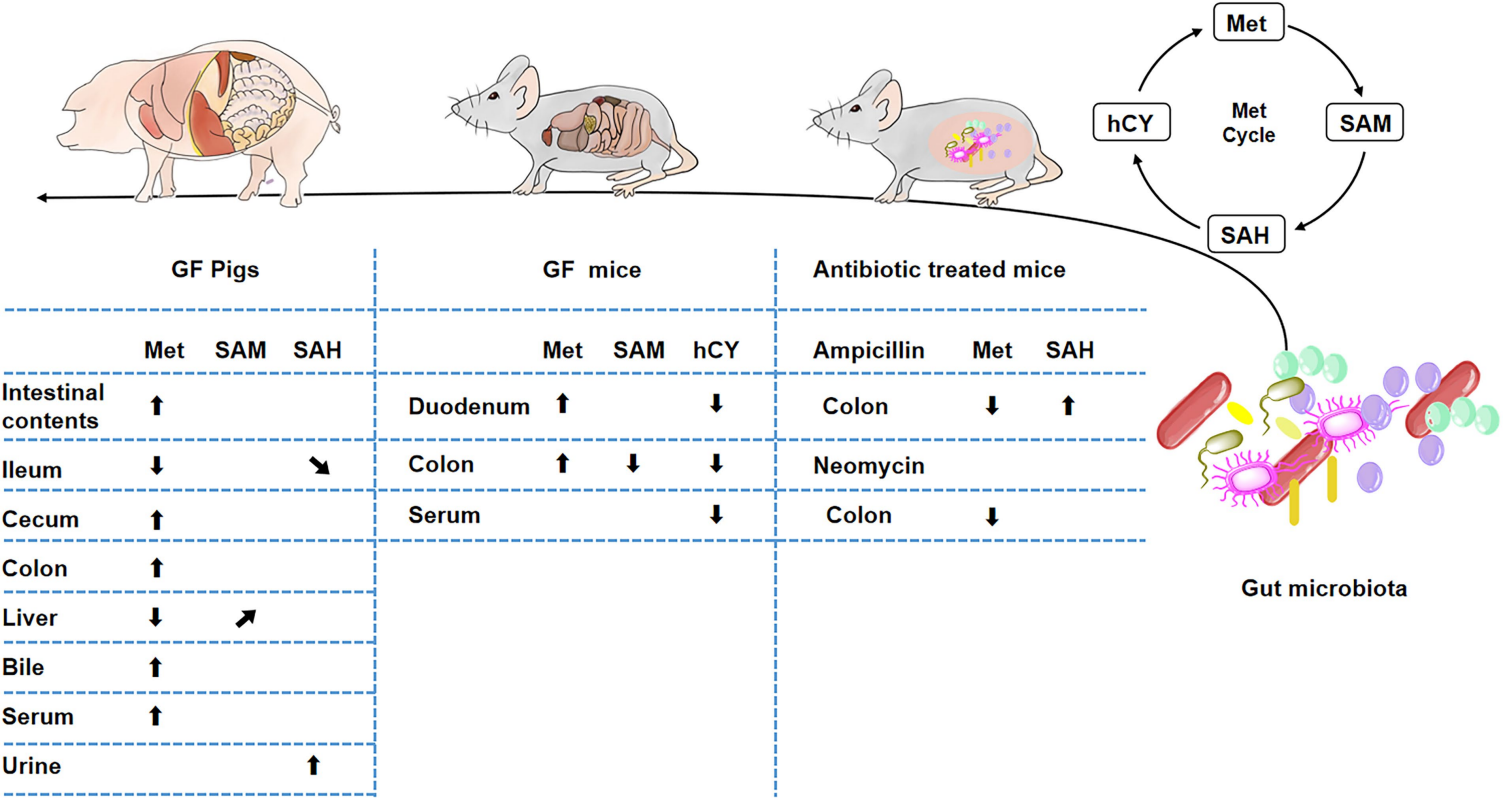
Influence of gut microbiota on Met metabolism. *(“⬆” arrows represent increases, “⬇” arrows represent decreases, “**↗**” arrows represent increasing trend, “**↘**” arrows represent decreasing trend).

Met metabolism is mainly composed of Met-cycle and produces the important methyl donor SAM, which participates in the biosynthesis of various proteins and DNA (Clare et al., 2019). SAM is demethylated to generate SAH and then SAH is deadenosine to generate hCY which may participate in the folate cycle and generate Cys (Lam et al., 2021). Cys produces metabolites, such as Tau or GSH, to regulate metabolic homeostasis (Selhub, 1999; Mentch et al., 2015; Jakubowski, 2019). The intestinal tract plays a key role in Met metabolism (Bauchart-Thevret et al., 2009). In addition to host intestinal transport or metabolism of Met (Zhang et al., 2014; Li et al., 2016; Romanet et al., 2021; To V et al., 2021), the gut microbiota also plays an important role in Met metabolism in the large intestine (Carbonero et al., 2012; Wang et al., 2019). Therefore, we used GF pigs as a model to explore the metabolites of the Met cycle and found that the gut microbiota was involved in regulating Met metabolism in host and the lack of gut microbiota led to the increase of Met in the gut and circulation system (Figure 8). Furthermore, we speculated that the liver is the main site of Met metabolism and utilization in GF pigs.

The increase of Met in the gut is mainly concentrated in large intestine, while the Met in small intestine shows a decreasing trend. We speculate that it might be due to the changes in intestinal Met transporters. Amino acid absorption in the gut relies on transport across the apical and basolateral membranes of epithelial cells. As a neutral amino acid (NAA), Met is taken up across the apical membrane of intestinal epithelial cells by various Na^+^-dependent/ independent transporters, such as *ASCT2* (*SLC1A5*), *B^0^AT1* (*SLC6A19*), *rBAT/b^0,+^AT* (*SLC3A1/SLC7A9*), *4F2hc/y^+^LAT1* (*SLC3A2/SLC7A7*), and *LAT4* (*SLC43A2*) primarily transports Met across the basolateral membrane (To V et al., 2021). Previous studies have demonstrated that the presence of *ASCT2* and *y^+^LAT1* in pig jejunum (Zhang et al., 2014; Li et al., 2016), *B^0^AT1* in pig jejunum and ileum (Yi et al., 2019; Hu et al., 2021), and *rBAT/b^0,+^AT* in pig ileum (Yi et al., 2019). *LAT4* is expressed in the human small intestine, especially the duodenum (Maric et al., 2021). A recent study has performed a segmented quantification of Met transporters in the porcine gastrointestinal tract and provided us with a more detailed description (Table 1). In addition to our previously mentioned Met transporters, *LAT2*, *IMINO*, *SNAT2*, and *ATB^0,+^* are expressed in the porcine gastrointestinal tract (Romanet et al., 2021). Moreover, the research found that in *Chd8*^+/−^ mice model of autism spectrum disorder (ASD), increased expression of neutral amino acid transporters *Slc6a19*, *Slc7a8*, and *Slc7a15* resulted in increased serum glutamine and tryptophan levels (Yu et al., 2022). In *Chd8*^+/−^ mice, the abundance of *Bacteroides* decreased, especially *Bacteroides uniformis*, and *Bacteroides* supplementation improved ASD-like behavior (Yu et al., 2022). In a piglet experiment, lysine restriction caused a decrease in *SLC7A1* and *SLC7A2*, limiting lysine transport (Yin et al., 2017). Sequencing found that lysine restriction increased the abundance of *Actinobacteria* and *Saccharibacteria* in the gut, and functional analysis of the microbial community revealed that the differences were concentrated in amino acid metabolism and membrane transport (Yin et al., 2017). Thus, gut microbiota has the ability to modulate different amino acid transporters. Therefore, we hypothesize that the gut microbiota might have varying degrees of influences on the Met transporters in the gut. Furthermore, based on the changes of Met-related metabolites in the gut and the expression locations of Met transporters, we suppose that *B^0^AT1*, *rBAT*, and *LAT4* might be partially inhibited, and/or *ATB^0+^*, *ASCT2*, and *SNAT2* might be promoted. However, this conjecture still needs to be verified by detecting the expression of related transporters. In addition, the gut microbiota has been shown to be involved in regulating intestinal mucosal integrity (Wang et al., 2019; Paone and Cani, 2020), angiogenesis (Franks, 2013; Andriessen et al., 2016), both of which are associated with nutrient absorption. Furthermore, gut microbes can utilize substances in the Met cycle (Carbonero et al., 2012; Wang et al., 2019; Das et al., 2021), or metabolize to produce Met-related downstream substances (Rosario et al., 2021), or interact with substances in Met cycle (Roth and Mohamadzadeh, 2021). Whether these functions of gut microbes are related to the regulation of host Met metabolism in this paper requires further experimental verification.

**Table 1:** mRNA Expression of Met transporters in different intestinal tracts of pigs.

Items	Duodenum	Proximal Jejunum	Middle jejunum	Ileum	Cecum	Proximal colon	Distal colon	References
*ATB^0+^*	H	L	L	L	H	H	H	Romanet et al. (2021)
*ASCT2*	M	M	M	M	M	M	H	Zhang et al. (2014), Romanet et al. (2021)
*B^0^AT1*	M	M	H	H	VL	VL	VL	Yi et al. (2019), Hu et al. (2021), Romanet et al. (2021)
*rBAT/b^0,+^AT*	H	H	H	H	L	VL	L	Yi et al. (2019), Romanet et al. (2021)
*y^+^LAT1*	H	H	H	M	L	L	L	Li et al. (2016), Romanet et al. (2021)
*LAT4*	H	H	H	H	L	L	L	Maric et al. (2021), Romanet et al. (2021)
*LAT2*	M	M	H	M	L	L	M	Romanet et al. (2021)
*IMINO*	M	M	M	H	M	M	H	Romanet et al. (2021)
*SNAT2*	M	M	M	M	M	H	M	Romanet et al. (2021)

We artificially classify the expression of these Met transporters as: < 0.1: very low expression (VL), 0.1 ~ 0.5: low expression (L), 0.5 ~ 1: moderate expression (M), >1: high expression (H). Refers to the data in this article (Romanet et al., 2021).

According to the experimental results, we found that gut microbiota could regulate host Met metabolism, but the increase in Met caused by lack of gut microbes was not detected in the jejunum. The possible reasons are: (1) Jejunal microbial metabolism: Jejunal microbiota is mainly involved in the degradation of lipids and sugars (Lema et al., 2020), so amino acid changes are not obvious; (2) Jejunal microbial types: the abundance and diversity of microbiota in the jejunum are very low (about 10^4^ ~ 10^7^ CFU/ml), and are mainly facultative anaerobic bacteria, such as *Streptococcus* and *Lactococcus*, which are different from the large intestine (Martinez-Guryn et al., 2019; Seekatz et al., 2019); (3) Features of the jejunum: in order to facilitate the nutrient absorption, the jejunum is covered with a thin layer of loose mucus, and the intervening Paneth cells secrete antimicrobial peptides (AMPs; Guo et al., 2017; Lema et al., 2020). And the transit time is faster in the small intestine compared to the large intestine (Martinez-Guryn et al., 2019), so the environment of the jejunum is not conducive to the long-term colonization of most microorganisms.

In addition, we examined the Met cycle-metabolites in GF mice, further confirmed that gut microbiota depletion caused the increase of Met, which promoted its downstream metabolic responses to a decrease in hCY and SAM (Figure 8). A variety of gram-positive or negative bacteria are involved in Met metabolism (Carbonero et al., 2012), thus, we treated mice with five different antibiotics, including the beta-lactam antibiotic ampicillin, polymyxin antibiotic colistin, nitroimidazole derivative metronidazole, aminoglycoside antibiotic neomycin, and glycopeptide antibiotic vancomycin. However, we could not achieve a Met increase similar to GF-treated effects by treating mice with antibiotics to mimic microbe deficiency. In contrast, we found a decrease in intestinal Met in the ampicillin and neomycin-treated groups (Figure 8).

Ampicillin can prevent the synthesis of bacterial cell walls with the bacteriostatic and bactericidal effects, and its effect on Gram-positive bacteria is similar to that of penicillin. Gram-negative bacteria such as *Escherichia coli* are sensitive to ampicillin (Jõers et al., 2010), while *Pneumococcus pneumoniae* and *Pseudomonas aeruginosa* are not (Li et al., 2021; Brazel et al., 2022). Neomycin has a good bactericidal effect on Gram-negative bacteria, Gram-positive bacteria, and *Mycobacterium tuberculosis*, of which *Escherichia coli* is the most sensitive (Kadison et al., 1951; Bera et al., 2008). Combined with the experimental results, we could only conclude that the lack of certain ampicillin- or neomycin-susceptible microbes resulted in lower intestinal Met levels. Moreover, studies have shown that there are objective function differences between GF animals and SPF animals such as metabolism and immunity (Uzbay, 2019), and the elimination of a single type of microbe may cause the increase in others, thereby probably leading to more utilization of Met. Determining the key microbes in host Met metabolic remodeling requires specific inhibition of possible microbial species and detection of Met metabolites after recolonization in GF animals. In addition to the changes of Met metabolism, studies have shown that growth performance, nutrient digestibility, and skeletal muscle growth and development are affected in GF piglets (Qi et al., 2021; Zhou et al., 2021). Therefore, it may be that the effect of gut microbiota on Met metabolism in GF animals was caused by the changes of global growth characteristics to drive the regulation of nutrient metabolism. In contrast to antibiotic-treated mice, GF animals was sterile in terms of other organs. In view of recent findings, the diversity of lung microbiota has a certain correlation with the risk of bronchopulmonary dysplasia (BPD) in neonates, and the lung microbiota metagenome of BPD probably changed the host metabolome (Lohmann et al., 2014; Lal et al., 2018). Therefore, it is also possible that microbiota in lung and other organs are involved in metabolic regulation in host, resulting the change trend of intestinal Met in antibiotic-treated mice is opposite to that in GF mice in this study. In order to study the influence of other organ microorganisms on Met metabolism, it may be a feasible experimental idea to redetect the changes of Met metabolism by supplementing intestinal microbiota of GF animals with whole intestinal microbiota transplantation (WIMT; Li et al., 2020).

In conclusion, the gut microbiota was involved in host Met remodeling, and Met was increased in complete GF animals (GF pigs and GF mice). However, when the microbes were cleared with antibiotics, ampicillin and neomycin treatment instead resulted in a reduction in intestinal Met. Since Met metabolism is closely related to various inflammatory diseases (Borren et al., 2021), ALD, liver tumors (Kharbanda, 2013; Xu et al., 2020), CVD, CKD (Yang et al., 2016), and other diseases, precise regulation of Met metabolism would become an effective means to treat these diseases. Therefore, our study provided new insights into the prevention and/or treatment of the associated diseases by modulating gut microbes in animals and humans.

## Data availability statement

The data presented in the study are deposited in the Metabolights repository, and the accession number is MTBLS6154.

## Ethics statement

The animal study was reviewed and approved by the Laboratory Animal Ethical Commission of the South China Agricultural University [permit number SYXK (Guangdong) 2019–0136].

## Author contributions

WT and SL designed the research and analyzed data. DY, JS, and LG assisted in the establishment of GF pigs and GF mice. XW, ZH, and BL participated in antibiotic-animal experiments. XW and BL prepared the figures and draft the manuscript. XW, ZH, WT, and SL revised and approved the final manuscript. All authors contributed to the article and approved the submitted version.

## Funding

This study was supported by the National Key R&D Program of China (2021YFD1301100), and Sichuan Science and Technology Programs (2021JDYZ0001 and 2021ZDZX0009).

## Conflict of interest

WT is employed by Sichuan Animtech Feed Co., Ltd.

The remaining authors declare that the research was conducted in the absence of any commercial or financial relationships that could be construed as a potential conflict of interest.

## Publisher’s note

All claims expressed in this article are solely those of the authors and do not necessarily represent those of their affiliated organizations, or those of the publisher, the editors and the reviewers. Any product that may be evaluated in this article, or claim that may be made by its manufacturer, is not guaranteed or endorsed by the publisher.

## Footnotes

^1^ www.ebi.ac.uk/metabolights/MTBLS6154

## Abbreviations

*A. muciniphila*, *Akkermansia muciniphila*
ALD, Alcoholic liver disease
CH3-THF, N5-methyltetrahydrofolate
CKD: Chronic kidney disease
CVD, Cardiovascular disease
Cys, Cysteine
ESI, Electrospray ionization
GF, Germ-free
Gly, Glycine
GSH, Glutathione
HCD, High collision-induced dissociation
hCY, homocysteine
HHcy, hyperhomocysteinemia
IBD, Inflammatory bowel disease
MAT, Methionine adenosyltransferase
MAT1A, Methionine adenosyltransferase 1A
Met, Methionine
NAA, Neutral amino acid
PD, Parkinson’s disease
SAH, S-adenosine homocysteine
SAHH, S-adenosylhomocysteine hydrolase
SAM, S-adenosine methionine
Sar, Sarcosine
SCFAs, Short-chain fatty acids
Ser, Serine
SPF, Specific pathogen-free
Tau, Taurine
